# Smartic: A smart tool for Big Data analytics and IoT

**DOI:** 10.12688/f1000research.73613.2

**Published:** 2024-02-06

**Authors:** Shohel Sayeed, Abu Fuad Ahmad, Tan Choo Peng

**Affiliations:** 1 Faculty of Information Science and Technology, Multimedia University, Melaka, Melaka, 75450, Malaysia

**Keywords:** IoT, Big Data Analytics, Data Cleaning, Data Imputation, Feature Engineering

## Abstract

The Internet of Things (IoT) is leading the physical and digital world of technology to converge. Real-time and massive scale connections produce a large amount of versatile data, where Big Data comes into the picture. Big Data refers to large, diverse sets of information with dimensions that go beyond the capabilities of widely used database management systems, or standard data processing software tools to manage within a given limit. Almost every big dataset is dirty and may contain missing data, mistyping, inaccuracies, and many more issues that impact Big Data analytics performances. One of the biggest challenges in Big Data analytics is to discover and repair dirty data; failure to do this can lead to inaccurate analytics results and unpredictable conclusions. Different imputation methods were employed in the experimentation with various missing value imputation techniques, and the performances of machine learning (ML) models were compared. A hybrid model that integrates ML and sample-based statistical techniques for missing value imputation is being proposed. Furthermore, the continuation involved the dataset with the best missing value imputation, chosen based on ML model performance for subsequent feature engineering and hyperparameter tuning. K-means clustering and principal component analysis were applied in our study. Accuracy, the evaluated outcome, improved dramatically and proved that the XGBoost model gives very high accuracy at around 0.125 root mean squared logarithmic error (RMSLE). To overcome overfitting, K-fold cross-validation was implemented.

## Introduction

The Internet of Things (IoT) is reshaping communication with technologies and is becoming a vital part of the development of a smart environment dedicated to make our lives convenient and comfortable. ^1^ Several IoT application sectors like smart homes, smart cities, ^2^ smart healthcare, assisted driving, smart retail, and consumer goods like wearables and smartphones are already available. ^3^^–^^5^ IoT is built with electronics hardware, software, and connectivity, which enables device interaction and transfer of data. The IoT ecosystem generates massive amounts of data. This data could be analyzed to make business decisions, ^6^ predict consumer behavior, or to bring solutions to problems that might exist. ^7^ Big Data offers the solutions to handle various types of data on a large scale.

Big Data extends the possibility to conduct extensive and rich analyses utilizing a vast amount of data. ^4^^,^^8^ Standard data processing tools are limited in data management capacity, where Big Data goes beyond the capabilities of traditional database management systems (DBMS). ^9^ Big Data comprises a large volume of information that is complex (structured and unstructured) in nature. Data are often being generated in real-time and can be of uncertain provenance. ^10^ New Big Data technologies are being developed to meet the demands for processing massive amounts of heterogeneous data. Big Data management benefits are significant and sometimes far-reaching, and many companies have started operating with Big Data to translate a large amount of data into valuable insights. ^11^

**Figure 1.**
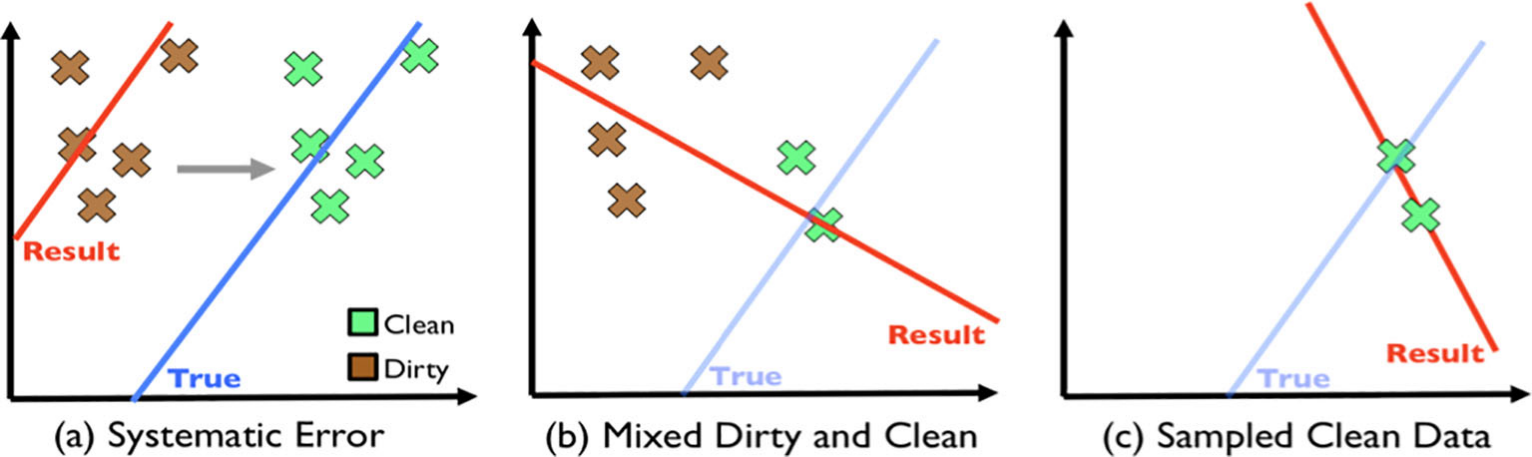
Simpsons Paradox.

The bulky and heterogeneous nature of Big Data requires investigation using Big Data Analytics (BDA). These data will yield meaningful outcomes by using methods of dissection in BDA, ^9^ which help to discover concealed patterns, anonymous relationships, trends of the current market situation, consumer preferences and other aspects of data that can assist institutes and companies to make up to date, faster and better decisions for their business. However, the biggest issue with available datasets is the data quality itself. The data quality issues differ depending on the data source; they could be duplicated records, spelling errors or more complex issues relating to unit misuse. A mixture of clean and dirty records in data can mislead to the well-known Simpsons Paradox ^12^^,^^13^ in which a pattern appears in a particular dataset but disappears or reverses when datasets are combined. A mixture of dirty and clean data could poorly fit an ML model; Figure 1 shows the different ML models fitting with different sets of mixed data. This would lead to unreliable analysis results. Hence, data pre-processing is an important factor in the data analysis process.

The data must be cleaned to make it suitable for analysis. Identifying dirty records and cleaning data sometimes require manual data inspection, which is time-consuming and can be costly. Pre-processing includes several steps, for example, (1) loading the data from the file, (2) cleaning it to fix inconsistencies or errors, (3) encoding the numeric and categorical data types, and finally, (4) the missing value imputation. Missing values can be handled in different ways. Columns or rows containing missing values can be dropped or a value can be imputed in each cell with a missing or improper value. Sometimes, crowdsourcing is used to correct some types of errors, which costs a significant amount of time and human-level works. Some researchers have used statistical computing such as mean, median, sum, among others, and approximate query processing to pre-process data. Some researchers have used sample-based cleaning techniques which can gradually improve data quality. Machine learning is an expanding research area, and is being used in some cases of data cleaning. A hybrid model of the data pre-processing technique called Smartic has been proposed by us, which has combined sample-based statistical techniques and ML. While sample-based statistical techniques lead to faster execution, ML models provide great accuracy. Our research contribution on Smartic will mitigate challenges related to dirty data cleaning and imputing missing values with better performance accuracy, within a reasonable time frame.

A tool for IoT data preparation and BDA with ML has been presented in this paper. Some feature engineering has been carried out after data pre-processing. This consists in checking which features are highly informative and which are less informative, and then considering features for the analytic purpose. Highly informative features will usually have the most benefits during feature development, while uninformative features can lead to overfitting. The main sections of this study are listed below:

• A review of the recent literature
• Presentation of a BDA framework
• Discussion of the data preparation issues and solution
• Presentation of techniques to improve analysis performance
• Comparison of different solutions and discussion of the results
• Conclusions and future research directions

## Related work

Ahmad *et al.* ^14^ reviewed the recent literature on IoT and BDA. Massive data production in IoT environments, and the versatile nature of the data, make Big Data a suitable solution for IoT systems. They discussed the opportunities for organizations to get valuable insights about their customers and help predict upcoming trends. BDA and ML ^15^ tools like classification, clustering and predictive modeling, provide data mining solutions that create many more opportunities to expose variability, improve decision-making habits and boost performance. ^16^ Cross-domain data gathered from different IoT appliances can be fed into BDA that can provide efficient solutions for different domains.

To overcome the challenges of collecting, processing, and examining the massive-scale, real-time data produced by smart homes, Bashir and Gill ^17^ offered an analytical framework composed of IoT, Big Data administration, and data analytics. The purpose of data analytics in their study was to automatically maintain the oxygen level consistency, detect hazardous gases or smoke, and control light conditions or quality. The work scheme was executed in the Cloudera Hadoop distribution platform, where PySpark ^18^ was used for big data analysis. The outcomes revealed that the proposed scheme could be used for smart building management with BDA.

Idrees *et al.* ^19^ proposed a two-step data cleaning method, using Big Data on a network of IoT wireless sensor devices. They attempted to minimize communication cost, save energy, and expand the lifespan of sensors by cleaning and reducing the redundant data. Their proposed two-level data reduction and cleaning approach in IoT wireless sensor networks includes a sensor level and an aggregator level. The aggregator level merged a near- similar data sets by implementing a divide and conquer technique. The reduced data sets were retransmitted to the sink, then the leader cluster algorithm-based cleaning method was applied to remove redundant data.

Salloum *et al.* ^20^ proposed a Random Sample Partition (RSP) Explore technique, to explore Big Data iteratively on small computing clusters. Their work included three main tasks: statistical estimation, error detection, and data cleaning. They partitioned the entire data into ready-to-use RSP blocks using an RSP-distributed data model. To get samples of clean data, they used block-level samples to understand the data and detect any potential value errors. Their experimental results showed that a sample RSP block cleaning is enough to get an estimation of the statistical properties of any dataset, and the approximate results from RSP-Explore can rapidly converge toward the true values.

García-Gil *et al.* ^21^ worked on data pre-processing to transform raw data into high-quality, clean data. The quality of the data used in any knowledge discovery process directly impacts the output. They experimented with classification problems due to the presence of noise affecting data quality, particularly a very disruptive feature of data known as incorrect labelling of training dataset. They proposed two Big Data pre-processing techniques with a special emphasis on their scalability and performance traits. The filters they used to remove noisy data were a homogeneous ensemble and a heterogeneous ensemble filter. The results from their experiments show that anyone can retain a smart dataset efficiently from any Big Data classification problem using these proposed filters.

Snineh *et al.* ^22^ proposed a solution that can be performed in real time to handle the frequent errors of Big Data flows. They proposed a repository for each given domain in their two-step model to store the metadata, cleaning and correction algorithms, and an error log. An advisor was appointed to supervise the system for the first step. The advisor could estimate the algorithm corresponding to error cleaning for a given context. At the second step, the system became autonomous in the selection algorithm procedure based on its learning module. That capability was obtained by using a strategy pattern-based approach. The pattern allowed the building of a family of algorithms, which are interchangeable and evolve independently of the context of use.

Jesmeen *et al*. ^23^ presented a comparison between currently used algorithms and their proposed tool, Auto-CDD, to handle missing values. The developed system improved overall data processing and guaranteed to overcome processing unwanted outcomes in data analysis. Their intelligent tool used Gini index values of random forest for feature selection. Experimental evaluation results showed that the random forest classifier led to a high accuracy on a diabetes dataset from UCI. ^24^ They also imputed the missing values on a student database and performed logistic regression analysis on students’ performance.

Shah *et al.* ^25^ investigated the research gaps in understanding Big Data characteristics generated by industrial IoT sensors, and studied the challenges to processing data analytics. They studied the characteristics of the Big Data generated from an in-house developed IoT-enabled manufacturing testbed. They explored the role of feature engineering for predicting the key process variables in effective machine learning models. The comparison with different levels or extent of feature engineering in between simple statistical learning approaches and complex deep learning approaches, shows potential for industrial IoT-enabled manufacturing applications.

El-Hasnony *et al.* ^26^ presented challenges in building an optimal feature selection model for Big Data applications, due to the complexity and high dimensionality of the data sets. They used particle swarm optimization and grey wolf optimization to build a new binary variant of a wrapper feature selection. The optimal solution was found with the help of the K-nearest neighbour classifier and Euclidean separation matrices. The overfitting issue was checked using K-fold cross-validation, and the performance and the effectiveness of the model were validated by conducting statistical analyses.

## Big data analytics

BDA follows some steps towards getting meaningful insights. Data analytics start with a non-trivial step of problem definition and evaluation. Research on expected gains and costs for reasonable solutions is needed. Generally, a data analytics framework is defined by five main steps:

### Data acquisition

Data acquisition, the key to the data life cycle, defines the data product profile. At this stage, structured and unstructured data are gathered from different sources and different types of unstructured or dirty data are pre-processed. Short data loading times are crucial for BDA due to its naturally exponential growth rate.

### Data mining and cleansing

The most essential stage of processing Big Data is to implement a method to extract the necessary data from the loaded, un-structured Big Data. A data analyst spends the most time on cleaning dirty data. Analysing dirty data could lead to erroneous results. To get high-quality data, faulty records, duplicates, unwanted records, and outliers need to be removed. Typos must be fixed and the data requires structuring. An exploratory analysis could investigate the initial characteristics of data and helps refining the hypothesis.

### Data aggregation and integration

The cleaned data obtained needs to be aggregated for processing numerical and categorical types of data, followed by data integration. Different types of data in various shapes and sizes obtained from different sources need to be integrated to prepare for analysis. The conversion between formats might be required for the unification of some data features. For example, one source collecting ratings on a five-star scale, and another source collecting data as “up” and “down” vote only. The response variable could be,

$$y\in \left\{1,2,3,4,5\right\}$$

and

$$y\in \left\{\text{positive},\text{negative}\right\}$$

Before the integration of both source data, an equivalent response representation needs to be created, possibly by converting the first source to the second representation format, considering three stars and above as the positive ratings and the rest of them as the negative ratings. Properly integrated data becomes less complex, more centralized and more valuable.

### Data analysis and modelling

From the perspective of Big Data, the goal is to produce meaningful insights that will be invaluable for business, through the analysis of data which may fluctuate depending on analytics technique and data types. Reports investigating the data must be constructed to help the business for better and faster decision-making.

### Data interpretation

Data interpretation allows to present data in an understandable format for users, for example, presenting data using analysis and modelling results to make decisions by interpreting the outcomes and extracting knowledge. Data interpretation queries are categorized together and indicate the same table, diagram graph or other data demonstration options.

## Proposed method

### Data collection and storage

Data has been collected from the UCI repository ^24^ and from publicly available datasets in the Kaggle database. ^27^ These datasets have been stored on Kaggle’s server, and they have been worked on within the kernels of the database. All collected datasets were in CSV (Comma-separated values) format.

### Data preprocessing

Data preprocessing is an important phase of data analysis. Raw data is manipulated to make it understandable. This is carried out in several steps, such as cleaning, encoding, imputing, among others. These steps have been handled separately.

### Data cleaning

**Figure 2.**
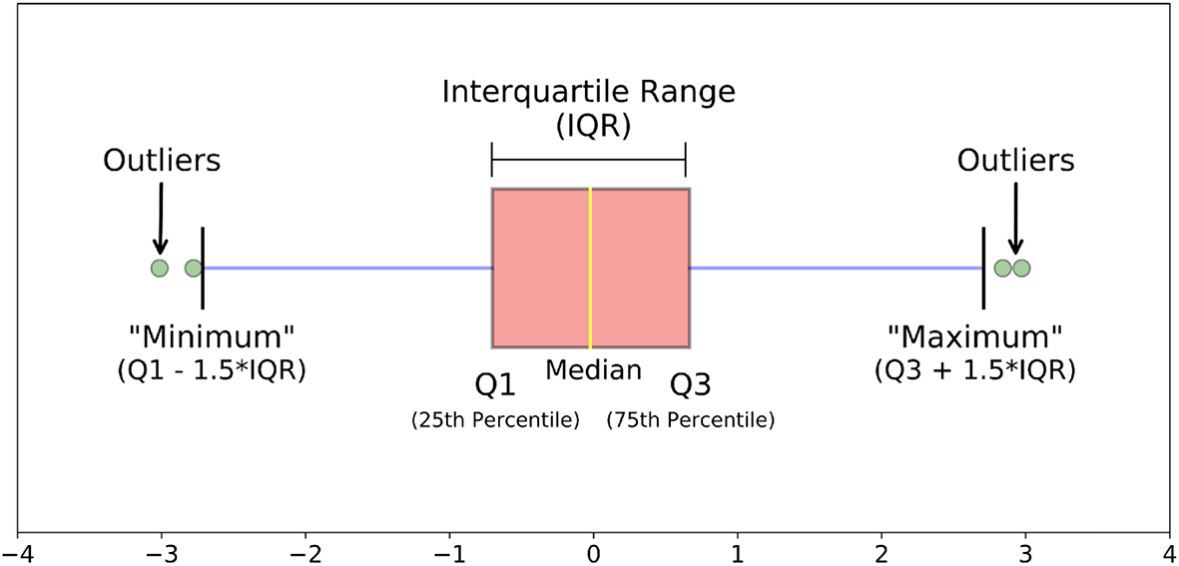
Outlier detection. ^28^

During this step, the focus has been on rectifying errors and removing inconsistency. Typos and various representations for values have been rectified into a common representation. Fuzzy matching or edit distance algorithms have been used to remove inconsistency. Outlier detection and removal help to get better accuracy. Figure 2 shows summary statistics (number summary) to represent data, such as, minimum, maximum, median, quartiles (Q1, Q3). The first quartile (Q1) is the middle value between the smallest value and the median (or the 50 ^th^ percentile, or Q2) of the dataset. A 25% portion of values in the dataset resides below the first quartile.

Interquartile Range,IQR=Q3−Q1

minimum=Q1−1.5∗IQR

maximum=Q3+1.5∗IQR

IQR, or midspread, or middle 50%, is the statistical dispersion equal to the range from lower quartile (25th percentile) to the upper quartile (75th percentile). The values that do not reside within the range of the minimum and maximum value are defined as outliers ( Figure 2).

### Data encoding

Numerical and categorical values have been prepared in the form of statistical data. The standard statistical types such as numeric and categorical had similar representations in Pandas ^29^ and Python (version 3.10). Each feature has been treated correctly by encoding each column as its respective type of data, which helps to apply transformation consistency in further analytical processes.

### Imputation

The missing values in this step have been addressed. 0 has been employed as the default value for missing numeric data, and ‘None’ has been utilized as the default value for missing categorical data. Different techniques have been employed to impute missing values, and the machine learning model has been trained by feeding these imputed datasets. The best imputation technique has been chosen based on the model performances, and it has been utilized for further analytical processes. The implemented algorithmic steps were as follow:

Step 1: Retrieve sample clean dataset ( ${\text{Dataset}}_{\text{clean}}$ ) from the original dataset, excluding missing/incomplete values as much as possible.

Step 2: Order Features ( ${\text{Features}}_{o}$ ) based on feature utility scores or mutual scores.

Step 3: Select top features from ${\text{Features}}_{o}$ and apply step 5.

Step 4: Select the rest of the features from ${\text{Features}}_{o}$ and apply step 6.

Step 5: For a given feature $F_{i}$ : label $F_{i}$ as the target and the rest of the column in ${\text{Dataset}}_{\text{clean}}$ as features, and train the ML model to obtain missing or incomplete values for the original dataset.

Step 6: For a given feature $F_{i}$ : calculate statistical parameters (mean or median) of the $F_{i}$ column in ${\text{Dataset}}_{\text{clean}}$ , and obtain missing or incomplete values for the original dataset.

## Feature engineering

**Figure 3.**
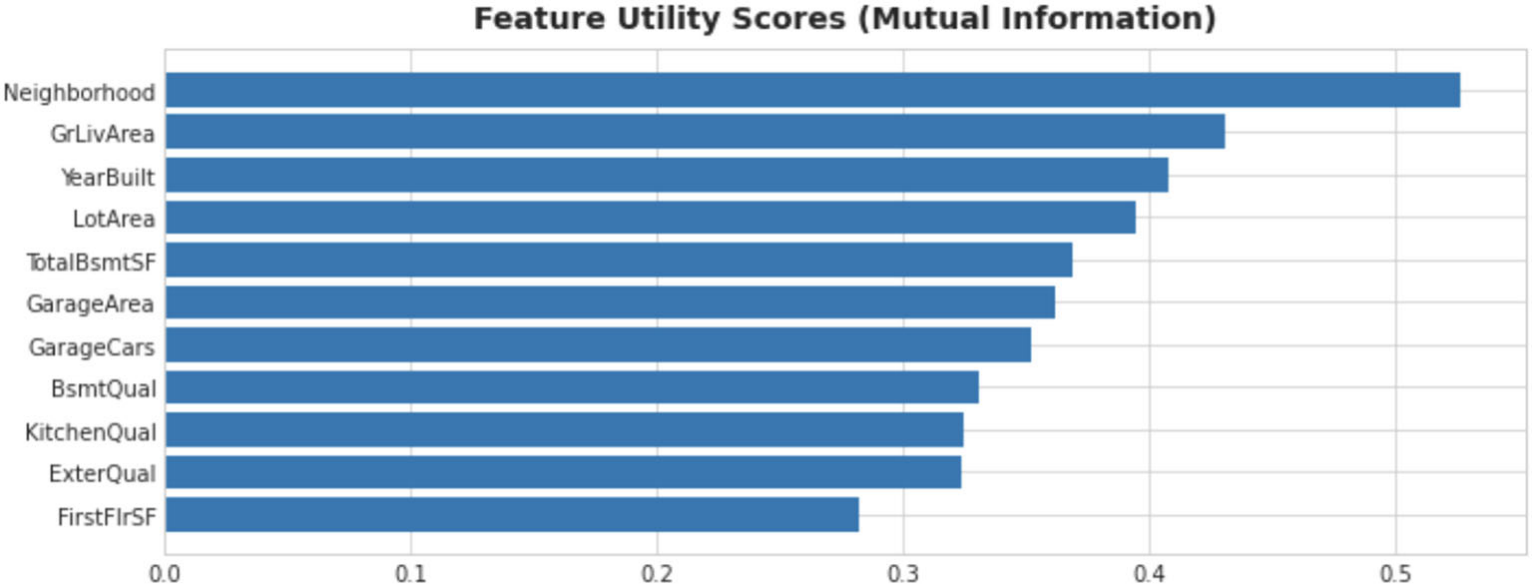
Feature utility scores of house prices dataset. ^27^

Mutual information ( Figure 3) has been employed to ascertain the importance of a feature. In this step, new features have been created as well. Target encoding has been utilized for categorical features with higher cardinality. Target encoding involves replacing a categorical feature with the average target value of all data points for that category. Several other techniques of feature engineering were used for this purpose.

### Feature utility scores

Using mutual score is a great way to determine a feature’s potential. Feature utility scores help to determine important features and non-important ones as well. Based on scores, some features have been discarded for a performance gain.

### Feature creation and transformation

Label encoding has been performed to transform categorical features, as the tree-ensemble model is the focus; this has been effective for both ordered and unordered data categories. Creating new features can be done in several ways such as, taking the product of two numerical features, the square root of a feature, normalize by applying logarithms, determining the group statistics of a feature, etc.

### K-means clustering

The unsupervised algorithm k-means clustering can be used to create features as well. Cluster labels or the distance of each entity to each cluster can be used as features. Sometimes, these help to untangle complicated relationships between features, engineered features or targets.

### Principal component analysis (PCA)

Another unsupervised principal component analysis (PCA) model has been applied for feature creation, leading to the decomposition of a variational structure. The PCA algorithm gave us loadings which described each component of a variation, and the components which were the transformed datapoints. Features may have been suggested to create by the loadings, and the components may have been directly used as features. Clustering can be done using one or more components.

### Target encoding

It is an encoding of categorical into numeric values derived from the target. It resembles a supervised feature engineering technique. The mean and median values have been used for this purpose.

### Hyperparameter tuning

A great way of boosting performance is carrying out hyperparameter tuning. A max_depth of 6, learning_rate of 0.01, and n_estimators of 1000 have been specified for our ML model XGBoost.

### Evaluation criteria

The K-fold cross-validation has been adopted for performance evaluation. The data set has been divided into training and testing data sets by cross-validation to train the model and assess its performance using two distinct data sets.

**Figure 4.**
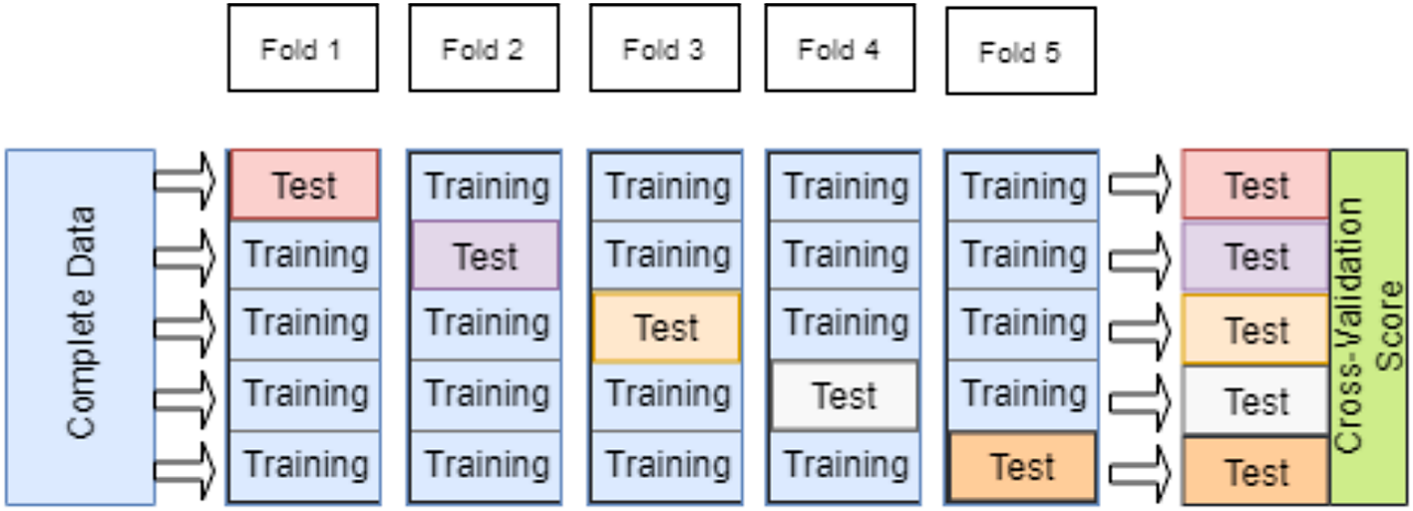
K-fold cross-validation (K=5).

The use of the same data for training and testing has led to the emergence of overfitting issues. To address this, K-fold cross-validation has been employed, with a K value of 5 ( Figure 4). All experimental results have been cross-validated using a five-fold approach. ^30^

The XGBoost model performance was evaluated using the root mean squared logarithmic error (RMSLE) metric. The formula for RMSLE is represented as follows:

$$\text{RMSLE}=\sqrt{\;\frac{1}{n}\;\sum_{i=1}^{n}{\left(\log\left(p_{i}+1\right)-\log\left(a_{i}+1\right)\right)}^{2}}$$

Where:

*n* is the number of observations in the dataset

$p_{i}$ is the prediction of target

$a_{i}$ is the actual target for *i*.

log( *x*) is the natural logarithm of *x* ( $\log_{e}\left(x\right)$ ).

## Experiments

### Discarding missing values

Discarding columns or rows is a technique for handling missing values. Our model performance in RMSLE was 0.14249 after discarding columns with missing values.

### Missing value imputation

Utilizing different imputation techniques, datasets with imputed missing values have been employed for the evaluation of the XGBoost model. A score of 0.14351 has been attained in the RMSLE when filling non-numerical (NAN, not a number) values with 0, whereas a score of 0.14348 has been obtained by filling missing values with the next valid value in the same column. An improvement in performance, indicated by an RMSLE score of 0.14157, has been achieved when missing values in a feature column are imputed using the statistical mean.

### Feature engineering

Improved performances have been obtained through the execution of feature transformation and target encoding based on feature utility scores. A better performance has been achieved through the utilization of K-means clustering and PCA. An RMSLE score of 0.14044 has been obtained.

### Hyperparameter tuning

**Figure 5.**
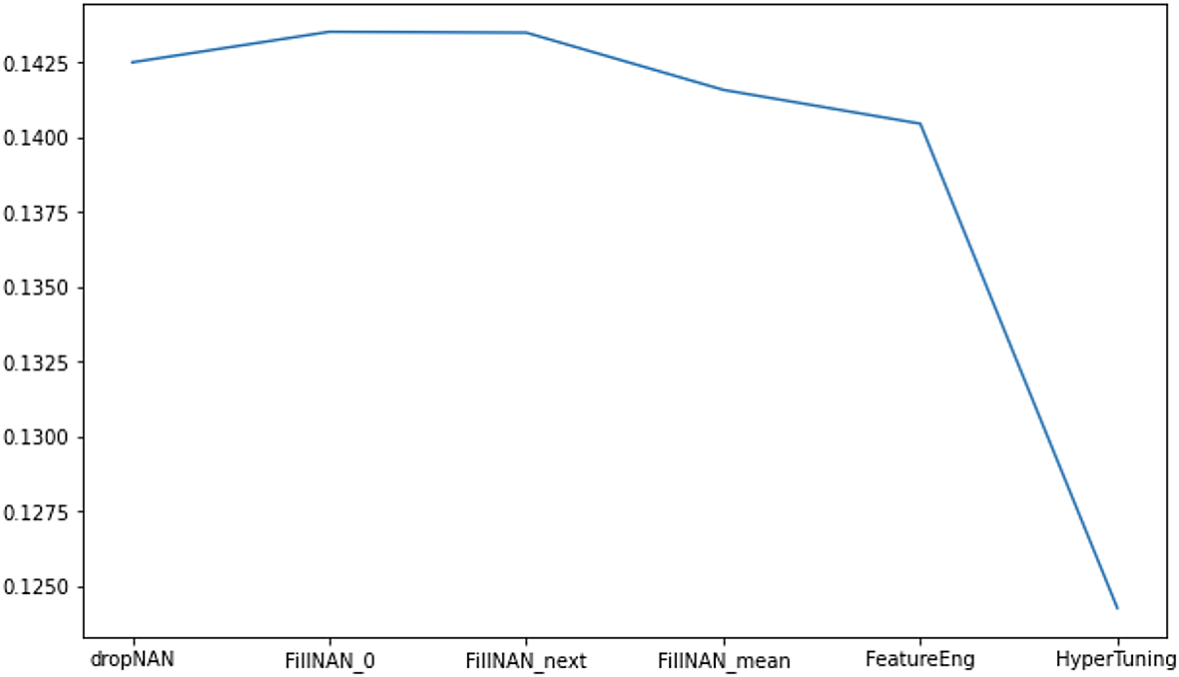
RMSLE metric score in different stage of analytics.

Hyperparameter tuning gave a performance boost in the final performance evaluation. Figure 5 shows the performance improvements after feature engineering and hyperparameter tuning.

A highest RMSLE score of 0.12426 has been obtained after the fine-tuning of some parameters.

## Results and discussion

**Figure 6.**
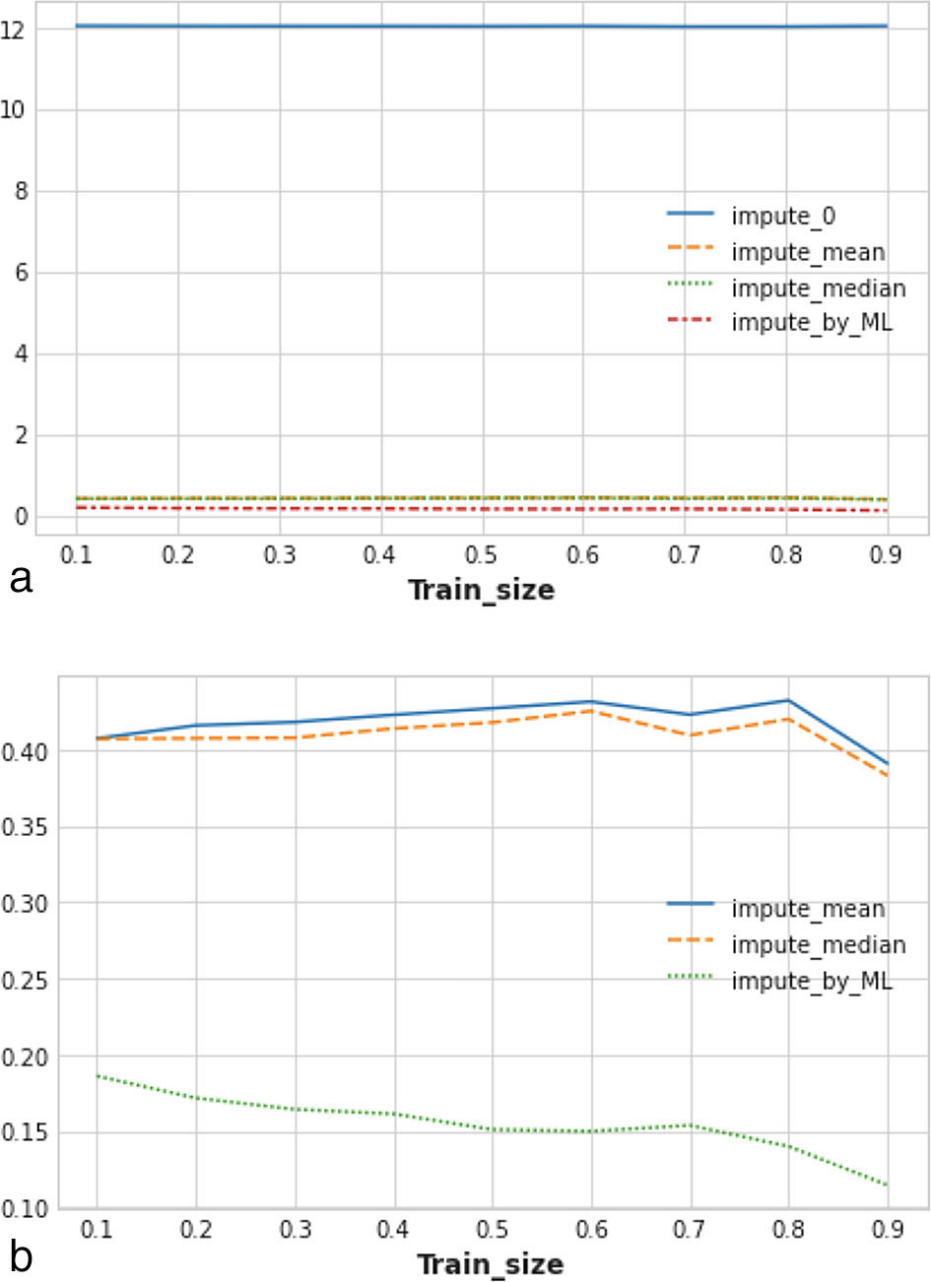
(a) Performance measurement of different techniques of missing value imputation. (b) Closer view of the best imputation techniques.

The mean RMSLE value has been calculated across five trials of train/test splits, with the training dataset size being varied from 0.1 to 0.9 (10% to 90%). In Figure 6(b), it has been observed that all other traditional imputation methods have been outperformed by the ML-based missing value imputation technique. Imputing 0 in place of the missing value performed worst in this experiment (see Figure 6a). Replacing missing values of any feature column with the median of that column performed slightly better than imputing the mean of that feature column.

**Figure 7.**
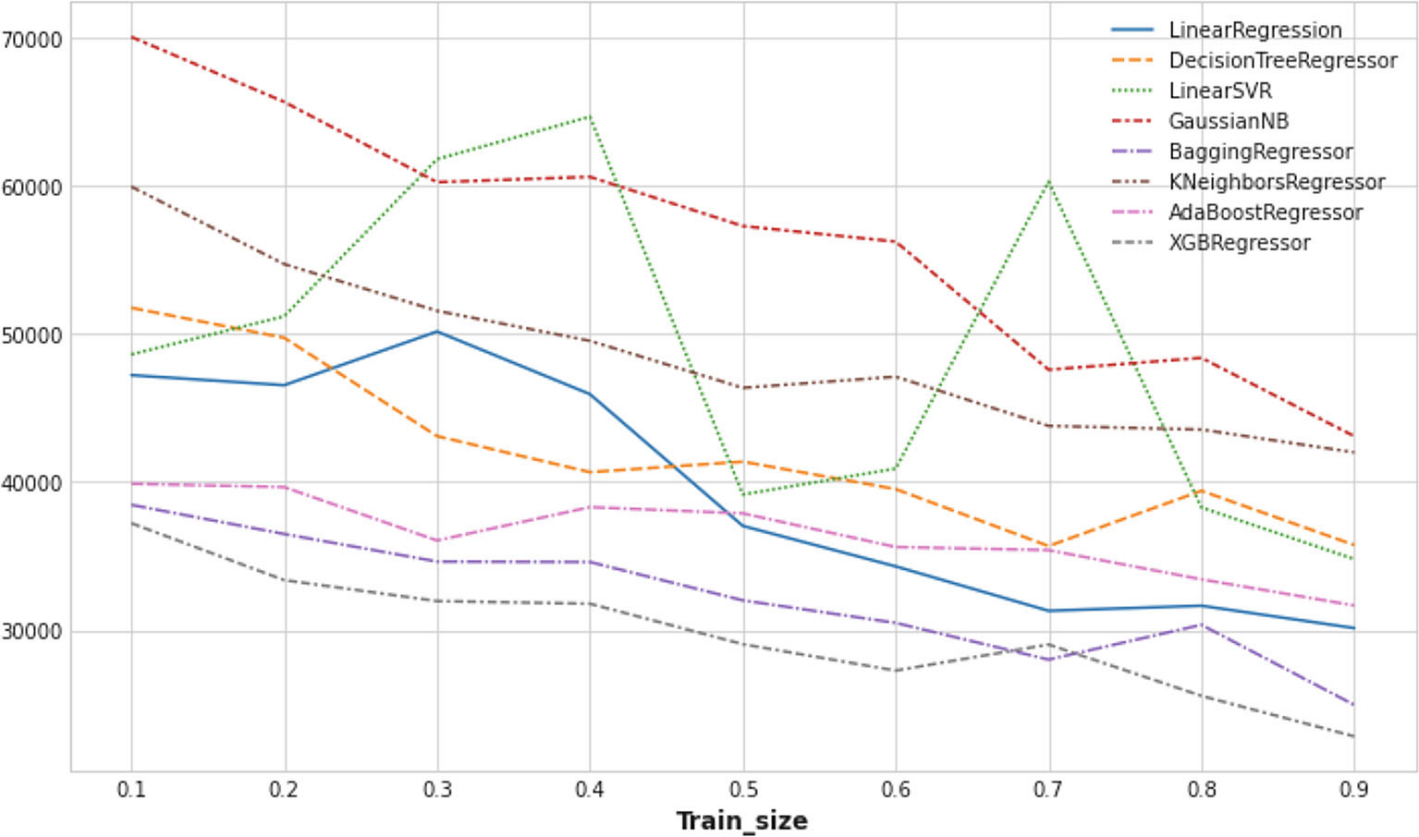
Mean squared error (MSE) of different machine learning (ML) models.

As the ML-based imputation technique outperformed state-of-the-art baseline methods. The evaluation of missing value imputation performance in different ML models has been conducted in this research such as LinearRegression, DecisionTreeRegressor, LinearSVR, GaussianNB, BaggingRegressor, KNeighborsRegressor, AdaBoostRegressor, XGBRegressor, among others. Although all ML models delivered higher accuracy with the continuous increment of training dataset size, more uniform and sheer increasing patterns have been noticed ( Figure 7) in models such as XGBRegressor and BaggingRegressor. It proves that with sufficiently large datasets, the XGBRegressor model can outperform the other ML methods. In addition, it has noticed that the XGBRegressor model showed a more stable performance with the varying training data size.

## Conclusion

Almost every data set available may contain missing values, which are essential to analyze and understand the data. Dealing with these types of dirty data is difficult, and getting a robust analytical ML models is more challenging. Statistical methods have been implemented to fix the datasets, and the integration of sample-based approximate query processing has been undertaken to alleviate errors in analysis and predictions. The data fixed using different imputation techniques were fed into ML analytical models, and accuracy was compared against different data preparation techniques. Smartic’s data value imputation was faster than the ML-based missing value imputation model. The ML model, trained on data cleaned using a sample-based technique, showed a significantly better and more stable performance. In the future, evaluation can be done with data collected directly from IoT environments in real time.

## Data availability

### Underlying data

- Ames housing dataset: house sales data in Ames, Iowa between 2006 and 2010. Compiled by Dean De Cock; used for educational purposes. The dataset used in this research is available at https://www.kaggle.com/c/house-prices-advanced-regression-techniques/data
- Diabetes dataset: The dataset represents clinical care at 130 US hospitals between years 1999-2008. This dataset was prepared to predict whether a patient’s re-admission. Dataset available from UC Irvine Machine Learning repository, https://archive.ics.uci.edu/ml/datasets/Diabetes+130-US+hospitals+for+years+1999-2008#

### Extended data

Analysis code available from: https://github.com/FuadAhmad/smartic

Archived analysis code at time of publication: https://zenodo.org/badge/latestdoi/420156995

License: (must be open access) Apache-2.0 License

## References

[1] Risteska Stojkoska BL Trivodaliev KV : A review of Internet of Things for smart home: Challenges and solutions. *J. Clean. Prod.* 2017;140:1454–1464. 10.1016/j.jclepro.2016.10.006

[2] Zanella A Bui N Castellani A : Internet of things for smart cities. *IEEE Internet Things J.* 2014;1:22–32. 10.1109/JIOT.2014.2306328

[3] Asghari P Rahmani AM Javadi HHS : Internet of Things applications: A systematic review. *Comput. Netw.* 2019;148:241–261. 10.1016/j.comnet.2018.12.008

[4] Hariri RH Fredericks EM Bowers KM : Uncertainty in big data analytics: survey, opportunities, and challenges. *J. Big Data.* 2019;6. 10.1186/s40537-019-0206-3

[5] Sepasgozar S : A systematic content review of artificial intelligence and the internet of things applications in smart home. *Appl. Sci.* 2020;10. 10.3390/app10093074

[6] Sestino A Prete MI Piper L : Internet of Things and Big Data as enablers for business digitalization strategies. *Technovation.* 2020;98:102173. 10.1016/j.technovation.2020.102173

[7] Ahmed E : The role of big data analytics in Internet of Things. *Comput. Netw.* 2017;129:459–471. 10.1016/j.comnet.2017.06.013

[8] Amalina F : Blending Big Data Analytics: Review on Challenges and a Recent Study. *IEEE Access.* 2020;8:3629–3645. 10.1109/ACCESS.2019.2923270

[9] Marjani M : Big IoT Data Analytics: Architecture, Opportunities, and Open Research Challenges. *IEEE Access.* 2017;5:5247–5261. 10.1109/ACCESS.2017.2689040

[10] Vikash LM Varma S : Performance evaluation of real-time stream processing systems for Internet of Things applications. *Futur. Gener. Comput. Syst.* 2020;113:207–217. 10.1016/j.future.2020.07.012

[11] Ge M Bangui H Buhnova B : Big Data for Internet of Things: A Survey. *Futur. Gener. Comput. Syst.* 2018;87:601–614. 10.1016/j.future.2018.04.053

[12] Fabris CC Freitas AA : Discovering Surprising Patterns by Detecting Occurrences of Simpson’s Paradox. *Research and Development in Intelligent Systems XVI.* 2000.

[13] Scarsini M Spizzichino F : Simpson-type paradoxes, dependence, and ageing. *J. Appl. Probab.* 1999;36:119–131. 10.1017/S0021900200016892

[14] Ahmad AF Sayeed MS Tan CP : A Review on IoT with Big Data Analytics. 2021. 10.1109/ICoICT52021.2021.9527503

[15] Al-Garadi MA Mohamed A Al-Ali AK : A Survey of Machine and Deep Learning Methods for Internet of Things (IoT) Security. *IEEE Commun. Surv. Tutorials.* 2020;22:1646–1685. 10.1109/COMST.2020.2988293

[16] L’Heureux A Grolinger K Elyamany HF : Machine Learning with Big Data: Challenges and Approaches. *IEEE Access.* 2017;5:7776–7797. 10.1109/ACCESS.2017.2696365

[17] Bashir MR Gill AQ : Towards an IoT big data analytics framework: Smart buildings systems. 2017. 10.1109/HPCC-SmartCity-DSS.2016.0188

[18] PySpark Documentation. Reference Source

[19] Idrees AK Jaoude CA Al-Qurabat AKM : Data reduction and cleaning approach for energy-saving in wireless sensors networks of IoT. 2020. 10.1109/WiMob50308.2020.9253429

[20] Salloum S Huang JZ He Y : Exploring and cleaning big data with random sample data blocks. *J. Big Data.* 2019;6. 10.1186/s40537-019-0205-4

[21] García-Gil D Luengo J García S : Enabling Smart Data: Noise filtering in Big Data classification. *Inf. Sci. (Ny).* 2019;479:135–152. 10.1016/j.ins.2018.12.002

[22] Snineh SM Youssfi M Bouattane O Daaif A : Real-Time management model for frequent Big Data errors: Automatic Clean Repository for Big Data (ACR). 2018. 10.1109/ICMCS.2018.8525920

[23] Jesmeen MZH : AUTO-CDD: Automatic cleaning dirty data using machine learning techniques. *Telkomnika (Telecommunication Comput. Electron. Control).* 2019;17:2076. 10.12928/TELKOMNIKA.v17i4.12780

[24] Strack B : Impact of HbA1c measurement on hospital readmission rates: Analysis of 70,000 clinical database patient records. *Biomed. Res. Int.* 2014;2014:1–11. 10.1155/2014/781670 24804245 PMC3996476

[25] Shah D Wang J He QP : Feature engineering in big data analytics for IoT-enabled smart manufacturing – Comparison between deep learning and statistical learning. *Comput. Chem. Eng.* 2020;141:106970. 10.1016/j.compchemeng.2020.106970

[26] El-Hasnony IM Barakat SI Elhoseny M : Improved Feature Selection Model for Big Data Analytics. *IEEE Access.* 2020;8:66989–67004. 10.1109/ACCESS.2020.2986232

[27] De Cock D : Ames, Iowa: Alternative to the boston housing data as an end of semester regression project. *J. Stat. Educ.* 2011;19. 10.1080/10691898.2011.11889627

[28] Galarnyk M : Understanding Boxplots.(accessed Oct. 21, 2021). Reference Source

[29] pandas. Reference Source

[30] Jacob W Sarah DP : k-Fold Cross-Validation Can Significantly Over-Estimate True Classification Accuracy in Common EEG-Based Passive BCI Experimental Designs: An Empirical Investigation. *Sensors.* 2023;23(13):6077. 10.3390/s23136077 37447926 PMC10346713

