# Supplementary material for: Smartic: A smart tool for Big Data analytics and IoT
Source: F1000Res. 2024 Feb 6;11:17. Originally published 2022 Jan 7. [Version 2] doi: 10.12688/f1000research.73613.2 (PMC10806368; doi:10.12688/f1000research.73613.2)
Supplement: Peer review history

# Reviewer report: Reviewer response for version 1

**DOI:** 10.5256/f1000research.77276.r136161

**Published:** 2024-01-23

**Authors:** Muzammil Hussain Mohammed

**Affiliations:** 1 Department of Information Technology, College of Computers and Information Technology, Taif University, Taif, Saudi Arabia

- I think this research paper well explains the content.
- The abstract is well defined.
- All the figures are clear and well understood.
- Related work is needed to add some more points to improve understanding. But overall it is ok.
- The proposed method is well explained with equations.
- Results and discussion are also explained good.

Overall this paper is well structured and good research work done by the authors.

Is the rationale for developing the new method (or application) clearly explained?

Yes

Is the description of the method technically sound?

Yes

Are the conclusions about the method and its performance adequately supported by the findings presented in the article?

Yes

If any results are presented, are all the source data underlying the results available to ensure full reproducibility?

Yes

Are sufficient details provided to allow replication of the method development and its use by others?

Yes

Reviewer Expertise:

Software Engineering, Cyber Security, Database and Networking

I confirm that I have read this submission and believe that I have an appropriate level of expertise to confirm that it is of an acceptable scientific standard.

# Reviewer report: Reviewer response for version 1

**DOI:** 10.5256/f1000research.77276.r136164

**Published:** 2022-05-30

**Authors:** Sujatha Krishnamoorthy

**Affiliations:** 1 Department of Computer Science, Wenzhou-Kean University, Wenzhou, China

I appreciate the authors for the work that has been carried out, as we are all dealing with the big data day to day in our life, which is very important. Big Data refers to large, diverse sets of information with dimensions that go beyond the capabilities of widely used database management systems, or standard data processing software tools to manage within a given limit. Almost every big dataset is dirty and may contain missing data, mistyping, inaccuracies, and many more issues that impact Big Data analytics performances. One of the biggest challenges in Big Data analytics is to discover and repair dirty data; failure to do this can lead to inaccurate analytics results and unpredictable conclusions. This experimented with different missing value imputation techniques and compared machine learning (ML) model performances with different imputation methods. The authors have proposed a hybrid model for missing value imputation combining ML and sample-based statistical techniques. The authors have used k-means clustering and principal component analysis. Accuracy, the evaluated outcome, improved dramatically and proved that the Boost model gives very high accuracy at around 0.125 root mean squared logarithmic error (RMSLE). To overcome overfitting, we use K-fold cross-validation.

 As per the quality of the work, the code was impressive and also the source is attached to paper, which is very useful for the people who would like to take up the research in this field.

 The only suggestion from my side is the flow of the paper - the authors have mentioned “we “in multiple places. Hence, I suggest them to change to passive, which can make readability of the paper clearer.

 The methods in the data analytics are explained in the section 3 and also the same is clearly explained in the way that is being implemented in the paper.

 The font in the imputation steps could be formatted to give a unified look. Add a proper citation to the K-fold cross-validation since the data is directly given from the experiment.

 Overall the paper structure is good enough for indexing.

Is the rationale for developing the new method (or application) clearly explained?

Yes

Is the description of the method technically sound?

Yes

Are the conclusions about the method and its performance adequately supported by the findings presented in the article?

Yes

If any results are presented, are all the source data underlying the results available to ensure full reproducibility?

Yes

Are sufficient details provided to allow replication of the method development and its use by others?

Yes

Reviewer Expertise:

IoT, Machine learning ,Computer vision

I confirm that I have read this submission and believe that I have an appropriate level of expertise to confirm that it is of an acceptable scientific standard.
